# Chemistry and Bioactivities of Six Tunisian *Eucalyptus* Species

**DOI:** 10.3390/ph15101265

**Published:** 2022-10-14

**Authors:** Habiba Kouki, Flavio Polito, Laura De Martino, Yassine Mabrouk, Lamia Hamrouni, Ismail Amri, Florinda Fratianni, Vincenzo De Feo, Filomena Nazzaro

**Affiliations:** 1 Faculty of Sciences, Bizerte, Zarzouna 7021, Tunisia; 2 Laboratory of Biotechnology and Nuclear Technology, National Center of Nuclear Science and Technology, Sidi Thabet, B.P. 72, Ariana 2020, Tunisia; 3 Department of Pharmacy, University of Salerno, Via San Giovanni Paolo II, 132, 84084 Fisciano, Italy; 4 Laboratory of Management and Valorization of Forest Resources, National Institute of Researches on Rural Engineering, Water and Forests, P.B. 10, Ariana 2080, Tunisia; 5 Institute of Food Science, CNR-ISA, Via Roma, 64, 83100 Avellino, Italy

**Keywords:** *Eucalyptus*, biodiversity, essential oils, phytotoxicity, biofilm

## Abstract

The complex taxonomy of *Eucalyptus* genus, the renewed interest in natural compounds able to combat microbial strains, the overuse of synthetic pesticides, the consequent request for alternative control methods were the reasons for this research. The essential oils (Eos) of *Eucalyptus bosistoana*, *Eucalyptus melliodora*, *Eucalyptus odorata*, *Eucalyptus paniculata*, *Eucalyptus salmonopholia,* and *Eucalyptus transcontinentalis* were analyzed by GC/MS and their potential phytotoxic activity was evaluated against the germination and radicle elongation of *Sinapis arvensis*, *Raphanus sativus* and *Lolium multiflorum*. The antibiofilm activity was assayed against both Gram-positive (*Staphylococcus aureus* and *Listeria monocytogenes*) and Gram-negative (*Pseudomonas aeruginosa, Escherichia coli,* and *Acinetobacter baumannii)* bacteria. Monoterpenoids were the most representative constituents in all EOs and eucalyptol was the dominant component except in *E. melliodora* EO, in which *p*-cymene was the most abundant. In phytotoxic assays, the EOs from *E. odorata* and *E. paniculata* were the most active against germination and radical elongation of the tested seeds. Finally, the *Eucalyptus* EOs proved their capacity to effectively inhibit the adhesion process of all five pathogen strains, with percentages often reaching and exceeding 90%. These *Eucalytpus* EOs could have possible employments in the food, health and agricultural fields.

## 1. Introduction

In 1788, the term *eucalyptus*, which means ‘well covered’, was coined by the French botanist, Charles Louis L’Héritier de Brutelle, referring to a genus with the operculate nature of the flower which lacks conspicuous petals and sepals [1].

Native to Australia, the genus *Eucalyptus* (Myrtaceae family) contains about 900 species and subspecies [2]. It has spread worldwide, particularly in Africa, because of its easy adaptability and fast growth [3] and it is now extensively cultivated in many countries for several applications such as cellulose, pulp, gum, essential oils, and honey production, as well as for construction and as an ornamental plant [4].

In order to improve the forest production, great efforts of reforestation based on *Eucalyptus* L’Hér species were implemented in Tunisia [3], where 117 species have been introduced. They were used as fire wood, for the production of mine wood, and in the fight against erosion [5].

*Eucalyptus* are woody perennial plants, varying from shrubs to tall trees: they can reach gigantic size and are mostly evergreen [6]. *Eucalyptus* species show leaf dimorphism: the juvenile leaves are opposite, oval to roundish, occasionally sessile and glaucous; mature leaves are alternate, entire, petiolate, lanceolate/elliptical/oblong/oval, often thick, stiff, highly cutinized and coriaceous [7]. Different types of inflorescence are observed within the species, axillary, umbel, cymes, panicles and corymbs; only in *E. globulus* Labill. solitary flowers are present. Fruits are woody capsule with seeds >1 mm to <2 cm in size, spherical, cuboid, elliptical, yellow to black colored [8].

*Eucalyptus* genus has a very complex taxonomic history. Different scholars have classified this genus and proposed various taxonomic positions time to time [6]: Brooker classified and combined *Eucalyptus* L’Hér. with *Angophora* Cav. and *Corymbia* Hill & Johnson in a single genus, *Eucalyptus*, but later several molecular studies and advanced phylogenetic analysis provided sufficient evidence to recognize both *Angophora* and *Corymbia* as separate genera [6].

The main products obtained from *Eucalyptus* are essential oils (EOs) [9], particularly employed for their antimicrobial [10], antifungal [11], antiseptic [12], wound healing, disinfectant [13] and phytotoxic abilities [2].

The leaves of over 300 species in this genus produce volatile oil [14]. The pharmaceutical and cosmetic industries have economically exploited only 20 species of *Eucalyptus* EOs rich in eucalyptol [15].

In recent years, the renewed interest in natural compounds is mainly due to those employed to combat microbial strains, exhibiting resistance to pharmacological substances [16]. Drug resistance develops among Gram-negative bacteria such as *Escherichia coli* and *Pseudomonas aeruginosa*, as well as Gram-positive bacteria like *Staphylococcus aureus* [17]. Different scientific works reported antimicrobial properties of the EOs from *Eucalyptus* leaves [18,19]. In particular, the antibacterial activity of the essential oils from leaves of *E. globulus* and *E. camaldulensis* Dehnh. was investigated against *E. coli* and S. *aureus* [20]. However, their ability to fight against the immature and mature biofilms of Gram-positive and Gram-negative bacteria, which allow for an increase of their virulence and a consequent greater resistance to conventional antibiotics, is poorly explored [21].

The current overuse of synthetic agrochemicals, causing environment and human health problems and pesticide-resistant biotypes, the onset of resistance phenomena and the agrochemicals’ withdrawal and restrictions (Directive 91/414/EEC, July 1993 and Regulation 1107/2009/EC, 2011) on a European and worldwide scale, are encouraging a decrease in their use and the need for alternative and “green” control methods [22].

Volatile allelochemicals derived from eucalyptus oils demonstrated also herbicidal activity against many weed species: specifically, volatile oils from *E. citriodora* Hook. and *E. nicholii* Maiden & Blakely showed phytotoxic effects, respectively, against hairy beggarticks (*Bidens pilosa* L.), green amaranth (*Amaranthus viridis* L.), nepal dock (*Rumex nepalensis* Spreng.) and wild tamarind [*Leucaena leucocephala* (Lam.) de Wit] [23], and against redroot pigweed (*Amaranthus retroflexus* L.), purslane (*Portulaca oleracea* L.) and Russian knapweed (*Acroptilon repens* (L.) DC) [24].

Other eucalyptus oils have also shown antigerminative effects [18], thus suggesting their possible further use as natural herbicides.

Nevertheless, the phytotoxic activity of eucalyptus oils can also cause damage to some crops, so the focus of the research is on the discovery of new selective herbicides in which the effect against weeds is maximized and towards crops is reduced.

Therefore, the variability of species in the *Eucalyptus* genus, its complex taxonomy, and the renewed focus on new natural antibacterial and herbicidal compounds were the reasons for conducting this research. The goals were (1) to study the variations in chemical compositions of the EOs from the leaves of Tunisian *Eucalyptus bosistoana* F. Muell., *E. melliodora* A. Cunn. ex Schauer, *E. odorata* Behr, *E. paniculata* Sm., *E. salmonopholia* F. Muell., and *E. transcontinentalis* Maiden; (2) to examine their potential phytotoxic activity and inhibitory effects against pathogenic biofilm.

## 2. Results

The hydrodistillation furnished pale yellow essential oils with variable yields: 1.0, 1.3, 0.6, 0.1, 3.0, and 1.6%, respectively, for *E. bosistoana*, *E. melliodora*, *E. odorata*, *E. paniculata*, *E. salmonopholia*, and *E. transcontinentalis*.

### 2.1. Chemical Composition of Essentials Oils

Table 1 reports the composition of the EOs. One hundred and four components were identified: the highest number (39) was identified in *E. bosistoana* EO; conversely, only 16 components have been detected in *E. melliodora* EO. Eucalyptol was the main component of all EOs (except *E. odorata* EO), with a percentage ranging between 44.9 (*E. paniculata* EO) and 78.1% (*E. melliodora* EO). α-Pinene was the second main component in *E. transcontinentalis* EO (14.8%), *E. salmonopholia* EO (10.9%) and *E. melliodora* EO (8.2%). *trans*-Pinocarveol is the second main compound of the oils of *E. bosistoana* (6.8%) and *E. paniculata* (10.8%). In *E. odorata* EO, p-cymene represented the main component (25.4%), followed by neo-verbanol (7.9%). Monoterpenoids were the main components in all EOs, mainly represented by oxygenated ones, ranging between 62.7 (*E. odorata* EO) and 89.0% (*E. melliodora* EO). Sesquiterpenoids ranged between 14.9 (in *E. salmonopholia* EO) and 13.0% (in *E. paniculata* EO), while they were absent in *E. melliodora* EO.

In 1987, Holeman and coworkers [25] found an *E. bosistoana* EO with a different chemotype, in which α-terpineol was the main component. Bouzabata and coworkers [26] also studied the composition and the chemical variability of eight EOs of *E. bosistoana* from Algerian Sahara, dividing the samples in two groups: in the first group, p-cymene was the dominant component; in the second group, as in sample here reported, high percentages of eucalyptol, ranging between 55.3 and 63.9%, were present. *E. bosistoana* and *E. melliodora* EOs, distilled from plants growing in Morocco region, had a chemical composition similar to our samples [27]. The compositions of EOs distilled from Tunisian *E. bosistoana* and *E. melliodora* were already reported in literature: Ameur and coworkers [14] found eucalyptol and α-pinene as the main components in both oils. These results partially confirmed the data reported in this work.

An EO from *E. melliodora* plants growing in Rwanda was reported by Umereweneza and coworkers [1] with a composition similar to the EO studied in this paper: eucalyptol and α-pinene were the most abundant components together with aromadendrene, not found in the studied sample. Sadeghi et al. [28] found an EO from *E. melliodora* with a similar composition: the authors reported eucalyptol and α-pinene with percentages of 51.1 and 9.5%, respectively. On the other hand, Eid and coworkers [29] reported a different constitution of an *E. melliodora* EO, with p-cymene (30.04%) and spathulenol (25.09%) as the predominant components.

The composition of *E. salmonopholia* essential oil was not previously reported in literature.

Elaissi et al. [5,30,31] reported chemical constitutions of an EO from *E. odorata* quite different from our data, with the ketone cryptone and p-cymene as the main components and a low amount of oxygenated monoterpenes.

However, the same authors [32] reported the composition of an *E. paniculata* EO with higher amounts (89.8%) of oxygenated components, first of all sesquiterpenes alcohols (τ-cadinol and 7-epi-α-eudesmol), sesquiterpenes oxides (caryophyllene oxide) and monoterpene oxides (eucalyptol). Dorsaf and coworkers [3] inferred the chemical characterization of a Tunisian EO from *E. transcontinentalis*; also in this case, the results displayed eucalyptol as the main component followed by α-pinene. Elaissi et al. [33] reported the composition of the EOs from several *Eucalyptus* species harvested in Tunisia, including *E. transcontinentalis*. This EO was characterized by a high amount of eucalyptol, followed by viridiflorol; in general, the results showed that the EO was rich in oxygenated sequiterpenes.

### 2.2. Phytotoxic Activity

The phytotoxic effects of the EOs, tested on germination and radical elongation of *Raphanus sativus* L. cv ‘Saxa’, *Sinapis arvenisis* L. and *Lolium multiflorum* Lam, were reported in Table 2.

The germination and radical elongation of radish were significantly inhibited by all doses of *E. bosistoana*, *E. odorata*, and *E. paniculata* EOs. Other EOs showed significant inhibitory effects on radish germination in the following order: *E. transcontinentalis* > *E. salmonopholia* > *E. melliodora*; whereas for the significant inhibitory effect of radish radical elongation the order was *E. transcontinentalis* > *E. melliodora* > *E. salmonopholia*.

The EO from *E. odorata* completely inhibited the germination of *S. arvensis*, at all doses tested. *E. paniculata* was the second most active oil; in fact, at the lowest doses tested, the inhibition of germination was 82.8% and the inhibition of radical elongation was 79.3%. The other EOs showed inhibition of germination >50% only at the two highest doses used. The EO of *E. salmonopholia* was the less active on radical growth of *S. arvensis*, while the other three oils showed inhibition of radical elongation of more than 50%, at all doses tested.

Only *E. odorata* and *E. paniculata* EOs totally inhibited the germination of *L. multiflorum*, at the highest doses tested. In the same case, *E. salmonopholia* and *E. melliodora* EOs seemed the less active EOs, with an inhibition of germination of 20.7 and 24%, respectively, at the highest doses tested. All other EOs (except *E. salmonopholia*) inhibited in a significant way the radical elongation of the seeds at all doses tested.

Recently, our research group reported the phytotoxicity and eco-compatibility of essential oils from *Eucalyptus gunnii* Hook. f. and *E. pulverulenta* Sims ‘Baby Blue’ cultivated in Italy [4]. The oils were tested on weeds (*L. multiflorum* Lam. and *Portulaca oleracea* L.) and crops (*Raphanus sativus* L., *Lactuca sativa* L., *Lepidium sativum* L., *Solanum lycopersicum* L., *Pisum sativum* L., and *Cucumis sativus* L.): both EOs inhibited *P. oleracea* seed germination, but only *E. pulverulenta* EO inhibited *L. multiflorum* radical elongation. Concerning crop species, the investigated EOs showed phytotoxicity in *R. sativus*.

Specifically, no reports on the phytotoxic activity of these EOs are present in the literature.

Several studies reported the potential herbicidal effect of the constituents of *Eucalyptus* EOs: Kaur et al. [34] studied the role of monoterpenes in *Eucalyptus* communities, highlighting the phytotoxicity/allelopathic activity of eucalypt EOs and of their monoterpenes constituents against germination and growth of many crops and weeds, as previously reported by many other authors [34,35,36,37,38].

Kordali et al. [39] investigated the inhibitory effects of monoterpenes on seed germination and seedling growth of different seeds and they concluded that, in general, oxygenated monoterpenes, especially alcohols, showed higher phytotoxic effects in comparison with monoterpene hydrocarbons, thus suggesting a potential use as bio-herbicides. In particular, De Martino et al. [40,41] reported that, between oxygenated monoterpenes, alcohols (borneol, citronellol, geraniol, α-terpineol) appeared as the most inhibitory compounds, followed by ketones (carvone, menthone, camphor) and aldehydes against germination of tested seeds. Alcohols and ketones were the most inhibitory mainly on radish radicle growth and monoterpene alcohol derivatives were more phytotoxic than their acetate derivatives [41]. The herbicidal effect could probably be related to the change in several biochemical and physiological processes, influencing the germination of seeds and/or the elongation of radicle hypocotyl [4]. However, as previously reported in the literature, *Eucalyptus* EOs can also cause damage to some crops, making it essential to assess their selectivity [4]. It is therefore critical to maximize the herbicidal activity of *Eucalyptus* against weeds but at the same time to minimize the negative impact on crop growth [2].

Zhou and coworkers [42] studied the phytotoxic and antimicrobial activities of some *Eucalyptus* essential oils, allowing to hypothesize a possible correlation between these effects. In fact, Khamassi et al. [21] suggested that the application of the essential oils on plant seeds during the germination process could generate oxidative stress, provoking a release of malondialdehyde from fatty acids of membrane phospholipids and so an alteration of membrane integrity, with relative leakage of electrolytes and loss of vital membrane functions. He et al. [43] suggested that when a microbial cell was treated with essential oils, the permeability of its membrane changed, producing a damage to a cell integrity and cell physiological functions.

### 2.3. Antibiofilm Activity

Table 3 shows the minimal inhibitory concentration of the *Eucalyptus* EOs necessary to inhibit the bacterial growth of the five pathogens used in our study.

MIC results allowed to evaluate, subsequently, the capacity of the six *Eucalyptus* EOs to influence the bacterial biofilm and their ability to affect the metabolism of the sessile bacterial cells. Such results could provide more information about the performance of EOs in limiting bacterial virulence which leads to increased bacterial resistance to conventional antibiotics. The results are shown in Table 4 and Table 5, respectively.

As reported in Table 3, the MIC values ranged between 26 and 32 µL/mL. Based on such results, two sub-lethal doses (10 and 20 µL/mL) were used in the antibiofilm tests.

The EOs proved their capacity to effectively inhibit the adhesion process of all five pathogenic tested strains. The inhibition was almost absolute in many cases, with percentages often reaching and exceeding 90% (Table 4). Except for the *E. melliodora* EO, which inhibited at 25.44% the adhesion of *A. baumannii*, the other EOs affected the biofilm formation with a powerful inhibitory action provoked by *E. transcontinentalis* EO (93.99%) and *E. odorata* EO (92.59%). Exciting results were also obtained by the *Eucalyptus* EOs against *E. coli*. In fact, some of these caused an inhibition even superior to 90% (*E. paniculata* and *E. transcontinentalis* EOs). The EOs, often already at a dose of 10 µL/mL, showed inhibitory efficacy *vs.* the bacterial adhesion of more than 80 percent compared to the control.

The experiments on *L. monocytogenes* (with percentages of inhibition ranging between 59.39 and 90.41%), *P. aeruginosa* (with inhibition ranges between 51.21 and 91.65%), and *S. aureus* (48.68–90.19%) indicate that all the EOs were capable of blocking, *ab origine*, the capacity of this strain to form a biofilm, the first step that leads to the increase of virulence. The anti-biofilm ability exhibited by the *Eucalyptus* EOs is of particular meaning. The pathogenic bacteria used in our experimentation play a particular role in the food and clinical fields: the common peculiarity was that they were often resistant to antibiotics, or at least increased their resistance due to the inappropriate use of conventional drugs. *A. baumannii* is widely considered one of the most threatening pathogens on a global scale, particularly in healthcare institutions, being one of the most difficult microorganisms to treat with conventional antibiotics and disinfectants [44]. The *A. baumannii* bacteremia could cause a significant increase in mortality with respect to other Gram-negative organisms [45]. It has been one of the “red alert” pathogenic bacteria that critically compromise the benefit of the presently applied antibacterial agents [46]. *E. coli* O157:H7 presents a legitimate concern for the public health [47], found in several foodstuffs, including milk and derivatives, meat and meat products, fruits and derivatives and green salads [48]. *P. aeruginosa* is a common Gram-negative able to adapt to adverse environments. It is capable of causing disease in humans, plants, and animals. Its connection with serious illnesses, especially hospital-acquired infections, indicated the difficulty of fighting such microorganism with standard drugs, also due to its capacity to form a biofilm [49]. Studies indicated that *L. monocytogenes* is the only species of the genus involved in known food-borne outbreaks of listeriosis, causing 10 to 40% mortality in patients [50]. *S. aureus* is an opportunistic pathogen, cause of nosocomial infections with high morbidity and mortality rates [51]. These aspects are also linked to its capacity to secrete multiple toxins and exoenzymes [52], and also to its ability to form biofilm [53]. These characteristics, together with its high multidrug resistance [54], determine a high degree of *S. aureus* pathogenicity.

The inhibitory activity exhibited by these EOs confirmed previous studies on these bacteria [21,55,56]. On the other hand, for first time, such powerful inhibitory effects have been noted on a range of Gram-negative and Gram-positive pathogens. This indicates that, while generally the non-specific resistance or sensitivity of the bacteria could be also depending on whether they are Gram-positive or Gram-negative bacteria [57], our results have indeed highlighted a wide potential applicability of these EOs. For example, the *E. transcontinentalis* EO, already at a dose of 10 µL/mL, caused an inhibition of not less than 79% (*vs*. *E. coli*) reaching 91.50% (vs. *A. baumanni*).

Comparing the ability of the *Eucalyptus* EOs to inhibit the adhesion process with the ability, in some ways even more important, to act on sessile cells present in the mature biofilm (thus capable of exhibiting even more pronounced virulence and more difficult to eradicate), the inhibitory efficacy was maintained, reaching inhibition percentages as high as 93.63 and 91.14% (*E. paniculata* EO *vs. L. monocytogenes* and *P. aeruginosa*, respectively). In some cases, an increase in inhibitory efficacy was evident: for example, *E. melliodora* EO increased its inhibitory capacities on mature *A. baumannii* biofilm from 25.44 to 36.37%. In each case, the inhibition was never less than 28.50%. This confirmed the inhibitory activity of the *Eucalyptus* EOs on the mature biofilm of different pathogens, including those used here, as indicated by other researches with the EOs of different *Eucalyptus* species [58,59,60,61].

As indicated in Table 4 and Table 5, the inhibitory effect of the *Eucalyptus* EOs could be ascribable mainly to their influence on the metabolism of the bacterial cell, particularly in the case of the EO of *E. odorata*. This EO determined an inhibition of the adhesion process of 88.56% and concurrently was capable of affecting the metabolism of the sessile cells at 83.36%. Only when the *E. salmonopholia* EO was tested against *P. aeruginosa*, the activity was extremely weak or absent. Therefore, the inhibitory effect of this oil, which produced an inhibition on the immature biofilm at 77.72%, could be due to other mechanisms such as the action on bacterial cells, DNA, bacterial permeabilization system, and others [57]. The effect of the EOs against the sessile cell metabolism within the mature biofilm indicated that often they did not act against the metabolic pathways of the bacterial cells. It happened when *E. melliodora* EO was added to the mature biofilm of *A. baumanni* and *L. monocytogenes*, or when *E. odorata* EO was administered to the mature biofilm of *E. coli*, *P. aeruginosa*, and *L. monocytogenes*. Again, the biofilm inhibitory action could be triggered by factors other than metabolic ones, also considering the different bacterial physiology in the mature biofilm. With the just mentioned exceptions, the test conducted using MTT showed that the *Eucalyptus* EOs were capable of acting on sessile cell metabolism even in the mature biofilm, reaching inhibition rates as high as 91.14% (*E. paniculata vs. P. aeruginosa*), 88.75% (*E. paniculata vs. E. coli*), and 85.95% (*E. paniculata vs. A. baumannii*). Furthermore, in all cases when the EOs worked, the inhibition rate never fell below 28.15% (*E. odorata vs. L. monocytogenes*) and 28.50% (*E. melliodora vs. A. baumannii*).

## 3. Materials and Methods

### 3.1. Plant Material

The leaves of *Eucalyptus melliodora, E. paniculata*, and *E. transcontinentalis* were collected at Henchir Naam ( 36.13° N, 9.10° E, 450 m alt.), an upper and middle semi-arid region (Tunisia). *E. odorata* was collected from Souiniet arboretum (35.54° N, 8.48° E, 492 m alt.), Jendouba, a region characterized by humid climate in the north part of Tunisia. *E. bosistoana* and *E*. *salmonopholia* leaves were collected from Djebel Mansour (36.16° N, 9.42° E), an upper and middle semi-arid region of Tunisia. The plants were identified by Professor Dr. Hamrouni Lamia and voucher specimens of the plants were kept in the herbarium division of the National Institute of Researches on Rural Engineering, Water, and Forests, Tunisia, labelled as EMA2105, EPA2109, ETCS2103, EBO2108, and ESA2102, respectively.

### 3.2. Extraction of Essential Oils

The leaves collected in April–May 2021 were reduced to fragments and then subjected to hydro-distillation for 3 h as reported in the *European Pharmacopoeia* [62]. The EOs were dissolved in *n*-hexane, dried over anhydrous sodium sulfate and kept under N_2_ at 4 °C in the dark until analysis.

### 3.3. Analysis of Essential Oils

Analytical gas chromatography was conducted on a Perkin–Elmer Sigma-115 gas chromatograph accessorized with an FID and a data handling processor. The separation was obtained with a HP-5MS fused-silica capillary column (30 m × 0.25 mm i.d., 0.25 μm film thickness). Column temperature: 40 °C, with 5 min initial hold, and then to 270 °C at 2 °C/min, 270 °C (20 min); splitless injection (1 μL of a 1:1000 *n*-hexane solution). Injector and detector temperatures were 250 °C and 290 °C, respectively. Analysis was also run by using a fused silica HP Innowax polyethylenglycol capillary column (50 m × 0.20 mm i.d., 0.25 μm film thickness). In both cases, He was employed as carrier gas (1.0 mL/min).

GC–MS analyses were conducted with a Hewlett–Packard 5890 A gas chromatograph linked on line to an HP mass selective detector (MSD 5970HP), equipped with a DB-5 fused-silica column (25 m × 0.25 mm i.d.; 0.33 µm film thickness). Ionization energy voltage 70 eV; electron multiplier energy 2000 V. Gas-chromatographic conditions were those described above; transfer line 295 °C.

Most components were identified by comparing their Kovats retention indices with those reported in the literature [63,64] or with those of authentic compounds available in our laboratory. The Kovats retention indices were calculated on the basis of a homologous series of *n*-alkanes (C_10_–C_35_) under the same operating conditions. Further identification was done comparing their mass spectra on both columns with either those present in NIST 02 and Wiley 275 libraries or with the literature [64,65,66], and in a personal library. Components’ relative concentrations were calculated by peak area normalization. Response factors were not considered.

### 3.4. Phytotoxic Activity

The phytotoxic activity of the EOs was evaluated on germination and root elongation of radish (*Raphanus sativus* L. cv ‘Saxa’), charlock mustard (*Sinapis arvensis* L.), and Italian ryegrass (*Lolium multiflorum* Lam) following the method previously reported [40]. These seeds are histologically well known and they are usually employed in phytotoxic assays for their fast germinability. The seeds were bought from Blumen group srl (Piacenza, Italy); their surface was sterilized in 95% ethanol for 15 s. The seeds were put in Petri dishes (Ø = 90 mm) with five layers of Whatman filter paper, impregnated with distilled water (7 mL, control) or the essential oil solution (7 mL) at different doses. The germination conditions were 20 ± 1 °C with natural photoperiod. The EOs and the pure compounds solubilized in water-acetone mixture (99.5:0.5) were tested at the doses of 1000, 500, 250, 125 µg/mL. No differences between controls performed with water–acetone mixture and controls with water alone were detected. Seed germination was checked directly in Petri dishes every 24 h. A seed was considered germinated when the protrusion of the root became evident [67]. After 120 h, the radicle lengths were measured and expressed in cm. Each determination was replicated three times, using Petri dishes containing 10 seeds each. The results were reported as the mean ± SD for both germination and radicle elongation.

### 3.5. Antibacterial Activity

#### 3.5.1. Microorganisms and Culture Conditions

*Acinetobacter baumannii* (ATCC 19606), *Escherichia coli* (DSM 8579), *Pseudomonas aeruginosa* (DSM 50071) (Gram-negative), *Listeria monocytogenes* (ATCC 7644), and *Staphylococcus aureus* subsp. *aureus* Rosebach (ATCC 25923) (Gram-positive) were the bacterial strains used in the experiments. Before the microbial analysis, bacteria were cultured in Luria Broth for 18 h at 37 °C (*A. baumannii* was grown at 35 °C) and 80 rpm (Corning LSE, Pisa, Italy).

#### 3.5.2. Minimal Inhibitory Concentration (MIC)

The resazurin microtiter-plate assay evaluated the MIC [68]. The tests were performed in flat-bottomed 96-well microtiter plates incubated at 37 °C and 35 °C, depending on the strain, for 24 h. The MIC value was revealed by the color change from dark purple to colorless. Determinations were performed in triplicate and results expressed as the mean ± SD.

#### 3.5.3. Biofilm Inhibitory Activity

The capacity of the EOs to influence the bacterial biofilm formation was evaluated in flat-bottomed 96-well microtiter plates (Falcon^®^, VWR International, Milano, Italy) [69]. Before the test, the overnight bacterial cultures were adjusted to 0.5 McFarland with fresh culture broth. Then, in each well 10 µL of the bacterial cultures, 10 or 20 µL/mL of each EO, and Luria–Bertani broth (LB, Sigma Aldrich Italia, Milano, Italy) were brought to a final volume of 250 µL. Microtiter plates were covered with a parafilm tape to preclude the evaporation of material included in the wells and incubated for 48 h at 37 °C (35 °C for *A. baumannii)*. Following the discard of the planktonic cells, sessile cells were lightly washed twice with sterile phosphate buffered saline (PBS), which was discarded, leaving the plates kept for 10 min under a flow laminar hood. Two hundred µL of methanol were included in each well, leaving it to act for 15 min to permit the fixation of the sessile cells. Methanol was discarded, and each plate was left to dry. The adhered cells were stripped by adding 200 µL of 2% *w*/*v* crystal violet solution to each well. After 20 min, the staining solution was discarded, and the plates were lightly washed with sterile PBS and left to dry. The bound dye was released by adding 200 µL of glacial acetic acid 20% *w*/*v*. The absorbance was measured at 540 nm (Cary Varian, Milano, Italy). The percent value of adhesion was calculated with respect to the control (formed by the cells grown without the presence of the EOs, for which the inhibition rate was assumed as 0%). Triplicate tests were done, taking the average results for reproducibility, and results were expressed as the mean ± SD.

#### 3.5.4. Inhibition of the Bacterial Metabolism

The effect of EOs on the metabolic activity of the sessile bacterial cells was evaluated through the 3-(4,5-dimethylthiazol-2-yl)-2,5-diphenyltetrazolium bromide (MTT) colorimetric method [69], using 96-well microtiter plates. The overnight bacterial cultures were adjusted to 0.5 McFarland, and the plates with 10 or 20 μl/mL of each EO and LB up to 250 μL were prepared as previously described. After 48 h of incubation in total, bacterial suspension representing the planktonic cells was removed, and 150 µL of PBS and 30 µL of 0.3% MTT (Sigma, Milano, Italy) were added, keeping microplates at 37 °C (35 °C for *A. baumannii*). The MTT solution was removed after 2 h, and the plates were washed twice with 200 µL of sterile physiological solution. Next, 200 µL of DMSO were added, leading to the formazan crystals’ dissolution, measured at 570 nm (Cary Varian, Milano, Italy) after 2 h. Triplicate tests were done taking the average results for reproducibility, and results were expressed as the mean ± SD.

### 3.6. Statistical Analysis

All assays were carried out in triplicate. Data of each experiment were expressed as the mean ± SD, and statistically analyzed by two-way ANOVA followed by Dunnett’s multiple comparisons test at the confidence level of 0.05 using GraphPad Prism 6.0 software.

## 4. Conclusions

The results agree with the previous literature regarding the phytotoxic properties of the *Eucalyptus* EOs, which could therefore represent an interesting green alternative in the herbicide scenario for use in agriculture. Furthermore, their demonstrated efficacy against the Gram-positive and Gram-negative bacteria used, often resistant to conventional antibiotics, could be related to their ability to decrease bacterial virulence and weaken the mechanisms of bacterial aggression, which is often the cause of the greater difficulty in eradicating the infections they are responsible for. Therefore, the activity exhibited by these *Eucalyptus* EOs against the pathogens such as *P. aeruginosa*, *S. aureus*, *E. coli*, *L. monocytogenes* and *A. baumannii* present in foods, workplaces and hospitals and responsible for several infections could be of considerable importance, both for food and health purposes. Moreover, a suggestive working hypothesis could orient the future research towards a possible link between phytotoxic and antibacterial activities.

## Figures and Tables

**Table 1:** Chemical composition of the EOs of *Eucalyptus bosistoana* (*Eb*), *E. melliodora* (*Em*), *E. odorata* (*Eo*), *E. paniculata* (*Ep*), *E. salmonopholia* (*Es*), and *E. transcontinentalis* (*Etr*).

KI ^a^	KI ^b^	Compound	*Eb*	*Em*	*Eo*	*Ep*	*Es*	*Etr*	Identification ^c^
770		*n*-Octane	t	-	-	-	-	-	1,2
864	1012	α-Pinene	1.1	8.2	1.5	1.5	10.9	14.8	1,2,3
876	1092	Camphene	t	0.2	0.1	-	0.1	0.1	1,2,3
883	1115	Thuja-2,4(10)-diene	t	-	-	-	-	-	1,2
902	1110	β-Pinene	-	-	2.5	3.4	0.5	0.4	1,2,3
903	1205	Limonene	t	-	-	-	-	-	1,2,3
922	1145	Myrcene	-	-	-	-	-	0.1	1,2,3
929	1177	α-Phellandrene	-	-	0.2	-	0.1	0.1	1,2,3
941	1170	α-Terpinene	-	-	0.3	-	-	-	1,2,3
952	1250	*a*-Cymene	-	-	25.4	1.6	2.1	-	1,2,3
957	1210	Eucalyptol	75.2	78.1	6.8	44.9	70.8	48.4	1,2,3
983	1221	γ-Terpinene	-	0.2	0.3	0.2	0.1	0.1	1,2,3
996	1115	*cis*-Sabinene hydrate	-	-	0.4	-	-	-	1,2,3
997		*cis* -Linalool oxide	-	-	-	0.1	-	-	1,2,3
1008	1291	Terpinolene	-	-	0.3	0.5	-	-	1,2,3
1010	1250	*p*-Cymenene	-	-	0.6	-	-	-	1,2
1011	1384	α-pinene oxide	t	-	-	-	-	-	1,2
1023	1506	Linalool	-	-	1.1	1.3	-	-	1,2,3
1029		Pentanoic acid, pentyl ester	-	0.3	-	-	0.7	0.3	1,2
1031		*exo*-Fenchol	0.2	0.6	-	-	-	0.5	1,2
1032		Isopinocampheol	-	-	-	-	0.3	-	1,2
1040	1474	*trans*-Sabinene hydrate	-	-	0.3	-	-	-	1,2
1042	1485	α-Campholenal	-	-	1.3	-	-	0.1	1,2
1043		3-Cyclopentene-1-acetaldehyd	-	0.1	-	-	-	-	1,2
1044		6-Camphenol	-	-	-	0.6	0.1	0.2	1,2
1051		Nopinone	-	-	0.5	-	-	-	1,2
1052	1382	*allo*-Ocimene	0.3	0.1	-	-	0.1	0.1	1,2,3
1055	1720	*trans*-Sabinol	-	-	3.6	-	-	-	1,2
1056	1753	Cumin aldehyde	-	-	2.4	1.2	-	-	1,2
1058	1664	*trans*-Pinocarveol	6.8	3.8	-	10.8	4,3	10.8	1,2
1062		*cis*-Pinene hydrate	-	0.1	-	-	-	-	1,2
1063	1663	*cis*-Verbenol	t	-	-	-	-	-	1,2
1065		*cis*-β-terpineol	-	-	0.5	-	-	0.1	1,2
1069	1643	Sabina ketone	-	-	7.7	1.1	-	-	1,2
1075		*trans*-Pinocamphone	-	-	-	-	-	0.1	1,2
1076	1468	*trans*-Limonene oxide	t	-	-	-	-	-	1,2
1078	1580	Pinocarvone	1.3	0.8	1.5	2.8	1.1	2.9	1,2,3
1082	1715	Borneol	0.3	1.2	1.3	2.2	0.2	0.6	1,2,3
1084		*neo*-Iso-isopulegol	-	0.2	-	-	-	-	1,2
**KI ^a^**	**KI ^b^**	**Compound**	***Eb***	***Em***	***Eo***	***Ep***	***Es***	***Etr***	**Identification ^c^**
1085	1832	*trans*-Carveol	0.2	-	-	-	-	-	1,2
1086		Menthol	-	-	-	-	0.1	-	1,2,3
1087		*cis*-Pinocamphone	-	-	-	-	-	0.2	1,2
1092		*cis*-Pulegol	t	-	-	-	-	-	1,2
1093	1590	Terpinen-4-ol	-	0.4	4	1.2	0.4	0.4	1,2,3
1097	1665	*neo*-Verbanol	-	-	7.9	1.8	-	-	1,2
1099	1678	*trans*-*p*-Mentha-2,8-dien-1-ol	0.9	-	-	-	-	-	1,2
1102	1661	α-Terpineol	-	3.3	6.7	-	0.8	0.8	1,2,3
1105	1828	*p*-Cymen-8-ol	-	-	1.7	-	-	-	1,2
1106		cis-Pinocarveol	-	-	-	-	0.3	0.1	1,2
1107	1678	*cis*-*p*-Mentha-2,8-dien-1-ol	0.1	-	-	-	-	-	1,2
1108		Myrtenal	-	-	-	-	-	0.5	1,2
1109	1661	β-terpineol	-	-	-	3.4	-	-	1,2,3
1110		Dihydro carveol	-	0.2	-	1.2	-	-	1,2
1111	1720	*trans*-Sabinol	t	-	-	-	-	-	1,2
1115	1791	Myrtenol	-	-	-	2	0.5	-	1,2
1122		*trans*-Dihydro carvone	-	-	-	-	-	0.2	1,2
1123	1726	Verbenone	-	-	-	1.5	-	-	1,2
1134	1683	*trans*-Verbenol	1.1	-	-	-	-	-	1,2
1142	1878	*trans*-Carveol	-	-	0.5	-	0.4	0.2	1,2
1150	1581	Thymol, methyl ether	-	-	7.4	-	-	-	1,2
1167	1717	Citronellol	-	-	-	-	0.2	-	1,2,3
1181	1720	*p*-Menth-1-en-7-al	-	-	4.6	-	-	-	1,2
1193	1491	Camphor	t	-	-	-	-	-	1,2,3
1198		α-Terpinen-7-al	-	-	-	0.2	-	-	1,2
1211	1868	Carvacrol acetate	-	-	-	0.5	-	-	1,2
1230	2172	Thymol	-	-	2.1	-	0.7	-	1,2,3
1247		*exo*-2-Hydroxycineole acetate		-	-	-	-	0.2	1,2
1248		Car-3-en-2-one	0.1	-	-	-	-	-	1,2
1248		γ-Terpinen-7-al	-	-	-	-	0.2	-	1,2
1250	2219	Carvacrol	-	-	-	-	0.3	-	1,2
1253		*trans*-Sabinene hydrate acetate	t	-	-	-	-	-	1,2
1327	1631	Aromadendrene	-	-	-	-	0.1	0.5	1,2,3
1332		Presilphiperfol-7-ene	0.8	-	-	-	-	-	1,2
1337		Silphinene	t	-	-	-	-	-	1,2
1349		Longicyclene	0.2	-	-	-	-	-	1,2
1361		α-Ylangene	t	-	-	-	-	-	1,2
1374	1660	*allo*-Aromadendrene	-	-	-	-	-	0.1	1,2,3
1384	1725	β-Selinene	-	-	-	-	-	0.1	1,2
1386	1548	β-Cubebene	0.1	-	-	-	-	-	1,2,3
1408		Viridiflorene	-	-	0.2	0.1	-	0.6	1,2
1433	1574	Longifolene	t	-	-	0.1	-	-	1,2
**KI ^a^**	**KI ^b^**	**Compound**	***Eb***	***Em***	***Eo***	***Ep***	***Es***	***Etr***	**Identification ^c^**
1434	1957	*epi*-Cubebol	-	-	-	0.2	-	-	1,2
1441		γ-Patchoulene	-	-	-	0.2	-	-	1,2
1444	2096	Epiglobulol	0.5	-	-	-	-	-	1,2
1448		α-Guaiene	0.4	-	-	-	-	-	1,2
1459	2127	Spathulenol	-	-	2.4	4.2	0.3	-	1,2
1460		α-Himalachene	1.6	-	-	-	-	-	1,2
1462		*cis*-Eudesma-6,11-diene	0.2	-	-	-	-	-	1,2
1464	2110	Viridiflorol	-	-	0.8	5.2	-	0.2	1,2
1465		(-)-Globulol	-	-	-	-	0.5	-	1,2,3
1466		Patchoulene	-	-	-	-	-	2.9	1,2
1467	1722	β-Selinene	3.5	-	-	-	-	-	1,2
1472		*trans*-β-Guaiene	-	-	-	0.1	-	0.4	1,2
1473	1748	*cis*-β-guaiene	0.8	-	-	-	-	-	1,2
1474	1752	γ-Cadinene	-	-	-	-	-	0.1	1,2
1475		10-*epi*-Cubebol	0.3	-	-	-	-	-	1,2
1482		Modhephen-8-β-ol	-	-	-	-	-	0.2	1,2
1483		allo-Cedrol	0.3	-	-	-	-	-	1,2
1495		Rosifoliol	0.2	-	-	-	-	0.2	1,2
1497		(E)-γ-Bisabolene	0.1	-	-	-	-	-	1,2
1501	2080	Cubenol		-	0.7	0.7	-	-	1,2
1505	2178	γ-Eudesmol		-	-	-	-	2	1,2
1515		β-Eudesmol		-	0.3	0.9	0.8	4.3	1,2
1523	2247	α-Eudesmol		-	-	1.3	-	3.3	1,2
		Total	96.6	97.8	97.9	97.0	97.0	97.2	
		Monoterpene hydrocarbons	1.4	8.7	30.3	6.7	13.9	15.7	
		Oxygentated monoterpenes	86.2	89.0	62.7	77.3	81.4	66.6	
		Sesquiterpene hydrocarbons	7.7	0	0.2	0.5	0.1	4.7	
		Oxygentated sesquiterpenes	1.3	0	4.2	12.5	1.6	10.2	
		Others	0.0	0.1	0.5	0.0	00	0.0	

^a^ Kovats index determined relative to the tR of a series of n-alkanes (C_10_-C_35_) on a HP-5 MS column; ^b^ Kovats index determined relative to the tR of a series of n-alkanes (C_10_-C_35_) on HP Innowax; ^c^ 1 = retention index reported in literature, 2 = mass spectrum, 3 = co-injection with authentic compound; t = trace <0.05%.

**Table 2:** Effects of different doses of the EOs on germination (number of germinated seeds) and radical elongation (cm) of *Raphanus sativus*, *Sinapis arvensis* and *Lolium multiflorum*.

*R. sativus* Germination						
Dose (µg/mL)	*E. bosistoana*	*E. melliodora*	*E. odorata*	*E. paniculata*	*E. salmonopholia*	*E. transcontinentalis*
Control	9.3 ± 1.2	9.7 ± 0.6	7.7 ± 0.6	8.0 ± 1.0	6.3 ± 1.5	8.0 ± 1.7
125	6.7 ± 1.2 **	9.3 ± 1.2	4.0 ± 1.0 ****	0.7 ± 0.6 ****	5.3 ± 2.5	6.3 ± 1.5
250	4.7 ± 0.6 ****	9.7 ± 0.6	1.3 ± 0.6 ****	0.7 ± 0.6 ****	5.7 ± 2.1	3.7 ± 0.6 ****
500	1.3 ± 0.6 ****	9.7 ± 0.6	0.3 ± 0.6 ****	0.0 ± 0.0 ****	1.7 ± 2.1 ****	1.3 ± 1.2 ****
1000	0.0 ± 0.0 ****	6.3 ± 1.5 ****	0.0 ± 0.0 ****	0.0 ± 0.0 ****	0.3 ± 0.6 ****	0.0 ± 0.0 ****
*R. sativus* Radical elongation						
Control	3.0 ± 0.1	6.3 ± 0.2	2.7 ± 0.3	3.2 ± 0.3	1.4 ± 0.1	4.0 ± 0.2
125	2.1 ± 0.3 ****	6.1 ± 0.4	1.7 ± 0.2 ****	1.0 ± 1.0 ****	1.0 ± 0.1	2.3 ± 0.6 *
250	1.6 ± 0.3 ****	4.1 ± 0.3 ****	0.6 ± 0.1 ****	0.4 ± 0.4 ****	1.1 ± 0.3	1.9 ± 0.5 **
500	0.6 ± 0.3 ****	3.1 ± 0.5 ****	0.1 ± 0.1 ****	0.0 ± 0.0 ****	0.6 ± 0.5	2.2 ± 2.0 *
1000	0.0 ± 0.0 ****	2.0 ± 0.1 ****	0.0 ± 0.0 ****	0.0 ± 0.0 ****	0.1 ± 0.2 *	0.0 ± 0.0 ****
*S. arvensis* Germination						
Dose (µg/mL)	*E. bosistoana*	*E. melliodora*	*E. odorata*	*E. paniculata*	*E. salmonopholia*	*E. transcontinentalis*
Control	10.0 ± 0.0	10.0 ± 0.0	9.7 ± 0.6	9.7 ± 0.6	10.0 ± 0.0	10.0 ± 0.0
125	9.3 ± 0.6	9.0 ± 1.0	0.0 ± 0.0 ****	1.7 ± 0.6 ****	9.7 ± 0.6	9.7 ± 0.6
250	4.0 ± 1.0 ****	5.3 ± 1.2 ****	0.0 ± 0.0 ****	0.3 ± 0.6 ****	6.7 ± 1.5 *	5.3 ± 1.5 ****
500	1.0 ± 1.0 ****	1.3 ± 0.6 ****	0.0 ± 0.0 ****	0.0 ± 0.0 ****	0.7 ± 0.6 ****	1.0 ± 1.7 ****
1000	0.0 ± 0.0 ****	0.3 ± 0.6 ****	0.0 ± 0.0 ****	0.0 ± 0.0 ****	0.0 ± 0.0 ****	0.0 ± 0.0 ****
*S. arvensis* Radical elongation						
Control	3.8 ± 0.1	6.5 ± 0.6	6.7 ± 0.2	3.9 ± 0.1	3.8 ± 0.8	3.8 ±0.8
125	1.3 ± 0.1 ****	2.4 ± 0.7 ****	0.0 ± 0.0 ****	0.8 ± 0.1 ****	2.5 ± 0.0 *	1.9 ± 0.7 **
250	0.6 ± 0.2 ****	1.2 ± 0.3 ****	0.0 ± 0.0 ****	0.1 ± 0.2 ****	1.4 ± 0.4 ****	0.5 ± 0.2 ****
500	0.7 ± 0.6 ****	0.7 ± 0.4 ****	0.0 ± 0.0 ****	0.0 ± 0.0 ****	0.6 ± 0.7 ****	0.4 ± 0.6 ****
1000	0.0 ± 0.0 ****	0.1 ± 0.2 ****	0.0 ± 0.0 ****	0.0 ± 0.0 ****	0.0 ± 0.0 ****	0.0 ± 0.0 ****
*L. multiflorum* Germination						
	*E. bosistoana*	*E. melliodora*	*E. odorata*	*E. paniculata*	*E. salmonopholia*	*E. transcontinentalis*
Control	9.3 ± 0.6	8.3 ± 0.6	8.7 ± 1.2	9.3 ± 0.6	9.7 ± 0.6	9.7 ± 0.6
125	9.0 ± 1.0	8.0 ± 1.0	7.3 ± 1.2	9.0 ± 1.0	9.7 ± 0.6	8.7 ± 0.6
250	8.7 ± 1.2	8.0 ± 1.0	6.3 ± 0.6 ****	8.7 ± 0.6	8.3 ± 0.6	8.0 ± 1.7
500	8.3 ± 0.6	7.3 ± 1.2	3.7 ± 0.6 ****	5.3 ± 0.6 ****	9.3 ± 0.6	8.0 ± 1.0
1000	4.7 ± 0.6 ****	6.3 ± 0.6	0.0 ± 0.0 ****	0.0 ± 0.0 ****	7.7 ± 0.6	1.0 ± 1.0 ****
*L. multiflorum* Radical elongation						
Control	4.7 ± 0.2	6.5 ± 0.2	5.4 ± 0.6	4.9 ± 0.2	5.1 ± 0.6	5.5 ± 0.3
125	3.2 ± 0.1 ****	3.8 ± 0.3 ****	1.6 ± 0.4 ****	2.7 ± 0.3 ****	5.5 ± 0.5	2.7 ± 0.2 ****
250	2.0 ± 0.2 ****	2.7 ± 0.2 ****	0.7 ± 0.3 ****	1.2 ± 0.2 ****	4.2 ± 0.7	1.6 ± 0.3 ****
500	0.9 ± 0.1 ****	1.3 ± 0.3 ****	0.3 ± 0.1 ****	0.3 ± 0.1 ****	3.5 ± 0.5 **	0.4 ± 0.2 ****
1000	0.5 ± 0.2 ****	0.5 ± 0.3 ****	0.0 ± 0.0 ****	0.0 ± 0.0 ****	0.8 ± 0.3 ****	0.1 ± 0.1 ****

Results are reported as the mean ± SD of three experiments. * *p* < 0.05, ** *p* < 0.01, **** *p* < 0.0001 *vs.* control, according to a two-way ANOVA followed by Dunnett’s post-hoc test.

**Table 3:** MIC (µL/mL) of the *Eucalyptus* EOs required to inhibit the growth of *A. baumannii*, *E. coli*, *L. monocytogenes, P. aeruginosa* and *S. aureus*. Tetracycline was used as a positive control.

	MIC (µL/mL)				
	*A. baumannii*	*E. coli*	*L. monocytogenes*	*P. aeruginosa*	*S. aureus*
*E. bosistoana*	27 ± 2	26 ± 2 *	28 ± 2	28 ± 1	24 ± 2
*E. melliodora*	30 ± 1 *	24 ± 1	30 ± 1 *	28 ±1	28 ± 1
*E. odorata*	28 ± 1	26 ± 1 *	30 ± 1 *	27 ± 1	27 ± 1
*E. paniculata*	26 ± 2	26 ± 1 *	28 ± 1	30 ± 2 *	28 ± 1
*E. salmonopholia*	30 ± 1 *	28 ± 1 ****	30 ± 1 *	30 ± 1 *	32 ± 1 ****
*E. transcontinentalis*	28 ± 1	26 ± 1 *	26 (±1)	28 ± 1	28 ± 1
Tetracycicline	27 ± 1	23 ± 1	27 (±2)	26 ± 2	26 ± 1

Data are expressed as the mean ± SD of three experiments. * *p* < 0.05; **** *p* < 0.0001 *vs*. Tetracycline according to a two-way ANOVA followed by Dunnett’s post-hoc test.

**Table 4:** Percent inhibition of bacterial biofilm formation caused by the addition of the *Eucalyptus* EOs at 0 and 24 h.

Time 0	*A. baumannii*	*E. coli*	*L. monocytogenes*	*P. aeruginosa*	*S. aureus*
*E. bosistoana* 10 µL/mL	59.40 ± 0.60 ****	68.52 ± 0.30 ****	57.71 ± 0.22 ****	63.26 ± 0.15 ****	65.76 ± 0.09 ****
*E. bosistoana* 20 µL/mL	76.69 ± 0.28 ****	78.83 ± 0.22 ****	58.77 ± 0.18 ****	74.77 ± 0.14 ****	69.39 ± 0.15 ****
*E. melliodora* 10 µL/mL	8.93 ± 0.78 ****	13.39 ± 0.64 ****	59.39 ± 0.22 ****	51.21 ± 0.29 ****	73.87 ± 0.08 ****
*E. melliodora* 20 µL/mL	25.44 ± 0.38 ****	30.35 ± 0.20 ****	73.31 ± 0.23 ****	60.28 ± 0.16 ****	76.40 ± 0.09 ****
*E. odorata* 10 µL/mL	88.56 ± 0.14 ****	86.26 ± 0.19 ****	78.83 ± 0.19 ****	75.39 ± 0.18 ****	82.48 ± 0.07 ****
*E. odorata* 20 µL/mL	92.59 ± 0.27 ****	88.09 ± 0.20 ****	79.57 ± 0.14 ****	77.72 ± 0.15 ****	85.77 ± 0.05 ****
*E. paniculata* 10 µL/mL	89.63 ± 2.51 ****	87.63 ± 2.45 ****	89.71 ± 1.03 ****	90.93 ± 0.58 ****	85.35 ± 0.76 ****
*E. paniculata* 20 µL/mL	93.01 ± 0.62 ****	90.89 ± 0.32 ****	91.65 ± 1.15 ****	93.50 ± 0.97 ****	87.02 ± 0.49 ****
*E. salmonopholia* 10 µL/mL	59.10 ± 0.15 ****	79.22 ± 0.08 ****	67.97 ± 0.20 ****	72.51 ± 0.11 ****	48.68 ± 2.06 ****
*E. salmonopholia* 20 µL/mL	66.06 ± 0.09 ****	85.00 ± 0.09 ****	69.88 ± 0.05 ****	81.41 ± 0.13 ****	58.45 ± 0.16 ****
*E. transcontinentalis* 10 µL/mL	91.50 ± 0.11 ****	79.69 ± 0.16 ****	89.25 ± 0.06 ****	83.74 ± 0.04 ****	87.72 ± 0.05 ****
*E. transcontinentalis* 20 µL/mL	93.99 ± 0.10 ****	91.02 ± 0.07 ****	90.40 ± 0.12 ****	83.89 ± 0.08 ****	90.19 ± 0.18 ****
Time 24 h					
*E. bosistoana* 10 µL/mL	39.70 ± 1.11 ****	39.23 ± 0.83 ****	17.69 ± 2.00 ****	45.62 ± 1.37 ****	78.63 ± 0.50 ****
*E. bosistoana* 20 µL/mL	58.01 ± 1.14 ****	61.40 ± 0.80 ****	46.21 ± 1.63 ****	60.36 ± 0.75 ****	79.84 ± 0.24 ****
*E. melliodora* 10 µL/mL	28.50 ± 0.54 ****	39.56 ± 0.37 ****	35.73 ± 0.68 ****	40.66 ± 0.75 ****	75.06 ± 0.04 ****
*E. melliodora* 20 µL/mL	36.37 ± 1.71 ****	46.42 ± 0.91 ****	37.44 ± 1.13 ****	56.57 ± 0.70 ****	80.01 ± 0.34 ****
*E. odorata* 10 µL/mL	56.52 ± 0.71 ****	33.71 ± 1.27 ****	28.15 ± 0.88 ****	49.83 ± 0.82 ****	80.05 ± 0.20 ****
*E. odorata* 20 µL/mL	59.10 ± 0.69 ****	58.53 ± 0.94 ****	33.73 ± 0.65 ****	54.66 ± 1.65 ****	80.63 ± 0.30 ****
*E. paniculata* 10 µL/mL	83.02 ± 2.27 ****	86.21 ± 2.54 ****	89.47 ± 1.71 ****	89.42 ± 0.61 ****	78.27 ± 0.76 ****
*E. paniculata* 20 µL/mL	85.95 ± 2.93 ****	88.75 ± 1.84 ****	93.63 ± 1.09 ****	91.14 ± 0.62 ****	85.38 ± 0.96 ****
*E. salmonopholia* 10 µL/mL	30.79 ± 1.83 ****	9.11 ± 0.91 ****	54.59 ± 0.60 ****	62.44 ± 0.69 ****	49.62 ± 0.33 ****
*E. salmonopholia* 20 µL/mL	51.03 ± 0.76 ****	62.63 ± 1.44 ****	63.61 ± 0.37 ****	64.50 ± 0.64 ****	53.76 ± 0.29 ****
*E. transcontinentalis* 10 µL/mL	38.45 ± 0.27 ****	27.15 ± 0.50 ****	40.29 ± 0.36 ****	60.57 ± 0.61 ****	79.55 ± 0.31 ****
*E. transcontinentalis* 20 µL/mL	47.47 ± 0.43 ****	46.80 ± 0.91 ****	41.69 ± 0.53 ****	60.63 ± 0.63 ****	81.29 ± 0.25 ****

Data are expressed as the mean ± SD of three experiments. **** *p* < 0.0001 *vs***.** control (0% of inhibition) according to a two-way ANOVA followed by Dunnett’s post-hoc test.

**Table 5:** Percent of inhibition on sessile cell’s metabolism induced by the presence of the EOs added at the beginning of the bacterial growth (time zero) and after 24 h.

Time 0	*A. baumannii*	*E. coli*	*L. monocytogenes*	*P. aeruginosa*	*S. aureus*
*E. bosistoana* 10 µL/mL	80.38 ± 1.48 ****	48.90 ± 1.96 ****	39.63 ± 2.20 ****	72.41 ± 2.17 ****	29.93 ± 0.92 ****
*E. bosistoana* 20 µL/mL	83.99 ± 0.81 ****	79.52 ± 1.83 ****	73.01 ± 2.20 ****	78.49 ± 0.77 ****	80.94 ± 1.85 ****
*E. melliodora* 10 µL/mL	66.85 ± 0.51 ****	0.00 ± 0.00 ns	77.69 ± 1.04 ****	70.89 ± 2.91 ****	78.41 ± 1.18 ****
*E. melliodora* 20 µL/mL	70.01 ± 0.75 ****	0.00 ± 0.00 ns	79.78 ± 0.28	75.46 ± 1.29 ****	80.71 ± 0.79
*E. odorata* 10 µL/mL	83.36 ± 0.67 ****	75.29 ± 1.48 ****	80.04 ± 1.28 ****	79.72 ± 1.18 ****	79.03 ± 2.67 ****
*E. odorata* 20 µL/mL	85.12 ± 0.70 ****	78.23 ± 3.98 ****	83.22 ± 0.97 ****	81.93 ± 0.37 ****	81.77 ± 0.30 ****
*E. paniculata* 10 µL/mL	50.43 ± 6.88 ****	68.65 ± 0.47 ****	76.13 ± 0.52 ****	69.05 ± 1.26 ****	81.24 ± 0.97 ****
*E. paniculata* 20 µL/mL	63.85 ± 4.11 ****	34.75 ± 0.89 ****	84.69 ± 0.22 ****	81.83 ± 1.42 ****	86.75 ± 1.65 ****
*E. salmonopholia* 10 µL/mL	0.00 ± 0.00 ns	0.00 ± 0.00 ns	64.05 ± 2.437 ****	0.00 ± 0.00 ns	48.08 ± 0.63 ****
*E. salmonopholia* 20 µL/mL	46.81 ± 2.00 ****	22.36 ± 1.88 ****	73.40 ± 1.30 ****	10.88 ± 2.09 ****	58.49 ± 0.63 ****
*E. transcontinentalis* 10 µL/mL	48.03 ± 3.60 ****	48.58 ± 1.76 ****	68.64 ± 1.84 ****	76.88 ± 2.60 ****	85.57 ± 1.35 ****
*E. transcontinentalis* 20 µL/mL	86.62 ± 2.14 ****	83.74 ± 3.00 ****	88.49 ± 1.73 ****	76.47 ± 3.08 ****	90.61 ± 1.83 ****
Time 24 h					
*E. bosistoana* 10 µL/mL	2.10 ± 1.75 ns	33.53 ± 0.39 ****	29.90 ± 1.09 ****	0.00 ± 0.00 ns	30.89 ± 1.52 ****
*E. bosistoana* 20 µL/mL	35.07 ± 1.51 ****	51.35 ± 0.92 ****	54.04 ± 0.88 ****	7.09 ± 0.44 ****	33.23 ± 0.52 ****
*E. melliodora* 10 µL/mL	0.00 ± 0.00 ns	16.49 ± 0.80 ****	0.00 ± 0.00 ns	4.17 ± 0.38 **	8.67 ± 0.68 ****
*E. melliodora* 20 µL/mL	0.00 ± 0.00 ns	38.93 ± 0.86 ****	0.00 ± 0.00 ns	17.09 ± 1.19 ****	18.98 ± 0.42 ****
*E. odorata* 10 µL/mL	0.00 ± 0.00 ns	20.60 ± 0.00 ****	0.00 ± 0.00 ns	0.00 ± 0.00 ns	7.95 ± 1.09 ****
*E. odorata* 20 µL/mL	0.00 ± 0.00 ns	58.69 ± 0.00 ****	0.00 ± 0.00 ns	0.00 ± 0.00 ns	28.29 ± 1.05 ****
*E. paniculata* 10 µL/mL	50.43 ± 6.88 ****	68.65 ± 0.47 ****	76.13 ± 0.52 ****	69.05 ± 1.26 ****	81.24 ± 0.97 ****
*E. paniculata* 20 µL/mL	63.85 ± 4.11 ****	34.75 ± 0.89 ****	84.69 ± 0.22 ****	81.83 ± 1.42 ****	86.75 ± 1.65 ****
*E. salmonopholia* 10 µL/mL	37.30 ± 0.52 ****	52.61 ± 0.45 ****	54.76 ± 0.47 ****	55.14 ± 0.80 ****	25.44 ± 1.74 ****
*E. salmonopholia* 20 µL/mL	39.33 ± 0.68 ****	72.10 ± 0.51 ****	56.57 ± 0.87 ****	60.74 ± 0.91 ****	58.72 ± 2.46 ****
*E. transcontinentalis* 10 µL/mL	60.94 ± 0.93 ****	67.46 ± 0.75 ****	57.14 ± 0.44 ****	66.75 ± 0.58 ****	42.86 ± 2.96 ****
*E. transcontinentalis* 20 µL/mL	65.08 ± 0.48 ****	70.81 ± 0.41 ****	60.60 ± 1.37 ****	72.90 ± 0.81 ****	55.87 ± 1.61 ****

Data are expressed as the mean ± SD of three experiments. ns: not significative; ** *p* < 0.01; **** *p* < 0.0001 *vs.* control (0% of inhibition) according to a two-way ANOVA followed by Dunnett’s post-hoc test.

## Data Availability

Data is contend within the article.
